# Plant Regeneration: Influencing Factors, Regulatory Networks, and Epigenetic Mechanisms

**DOI:** 10.3390/ijms27146259

**Published:** 2026-07-14

**Authors:** Wenke Song, Xin Cheng, Xinmin Liu, Limei Wang, Maoteng Li

**Affiliations:** 1 College of Life Science and Technology, Wuhan Polytechnic University, Wuhan 430023, China; swk1183@163.com; 2 Biological Seed Industry Research Institute, Xianghu Laboratory, Hangzhou 311231, China; 3 College of Life Science and Technology, Huazhong University of Science and Technology, Wuhan 430074, China; d202381022@hust.edu.cn (X.C.); d202381021@hust.edu.cn (X.L.)

**Keywords:** plant tissue culture, genetic transformation, plant hormones, developmental regulators, epigenetic reprogramming

## Abstract

Plant tissue culture is a crucial part of biotechnology that supports crop improvement, plant conservation, and other related fields. Although tissue culture has been successfully used in many plants, low regeneration and transformation rates still exist in some species. *Agrobacterium*-mediated transformation is commonly used in genetic engineering, but its effectiveness depends heavily on establishing a reliable in vitro regeneration system through organogenesis or somatic embryogenesis. Over the past decade, substantial progress has been made in understanding the molecular basis of regeneration; however, most reviews have focused on individual aspects such as hormone regulation or transcription factor networks in isolation. In contrast, this review provides a comprehensive and integrated framework that systematically links four critical layers—wound signaling, hormonal regulation, developmental regulators, and epigenetic modifications—into a unified regulatory network governing plant regeneration. Furthermore, we highlight recent cutting-edge advances, including artificial intelligence-assisted prediction, single-cell and spatial transcriptomics, epigenome editing, and CRISPR-based activation systems, and we discuss their transformative potential in overcoming genotype-dependent recalcitrance. By synthesizing classical regulatory mechanisms with emerging technologies, this review offers a forward-looking perspective that distinguishes it from earlier publications and provides both theoretical foundations and practical strategies for improving plant regeneration and genetic transformation.

## 1. Introduction

Plant tissue culture is a biotechnology tool that is widely used in both basic theoretical research and genetic improvement; it plays significant roles in studying plant developmental processes, analyzing gene function, creating transgenic plants, plant breeding and crop improvement, germplasm conservation, and preservation of vegetatively propagated crops, as well as rescuing rare and endangered plant species [1,2,3]. Meanwhile, transgenic technology combined with plant tissue culture is currently the mainstream approach for achieving crop genetic improvement, in which an efficient in vitro regeneration system is central to establishing an efficient genetic transformation platform. However, the capacity of in vitro regeneration in plants is highly dependent on species and genotype [4]. 

Among cereal crops, wheat has extremely low genetic transformation efficiency, and studies in recent years have shown that overexpressing *TaWOX5* can increase its transformation efficiency significantly [5]. In woody plants, regeneration efficiency is closely related to the age of the donor plant; for example, explants taken from 70-year-old pedunculate oak trees can induce root formation, whereas those taken from 600-year-old trees completely lose their root regeneration ability, and the number of regenerated shoots also significantly decreases [6]. Furthermore, genetic transformation and regeneration efficiency are consistently constrained by the genotype in some important economic crops. Taking soybeans as an example, mature explants exhibit very low regeneration rates, but using young tissues (such as immature cotyledons) can yield higher regeneration rates. Similarly, hemp regeneration shows high genotype dependence, and it was revealed that only one type successfully produced transgenic plants among 100 tested varieties [7]. Therefore, understanding the key factors affecting plant regeneration holds significant research importance for addressing the challenges of low efficiency in plant genetic transformation and regenerative capacity.

Despite the long history of plant tissue culture research and the publication of numerous reviews on this topic, most previous articles have focused on isolated aspects—such as individual hormone signaling pathways, specific transcription factor families, or epigenetic modifications in particular species—without providing a systematic integration of these regulatory layers [8,9,10,11,12]. Moreover, while some recent reviews have touched upon the molecular mechanisms of regeneration, few have comprehensively connected the initial wound-triggered signaling events to the downstream hormonal balance, transcriptional reprogramming, and epigenetic dynamics that collectively orchestrate cell fate transitions. Additionally, the rapid development of emerging technologies—including single-cell multi-omics, spatial transcriptomics, AI-based predictive modeling, and CRISPR-based epigenome editing—has dramatically reshaped the research landscape, yet these advances have not been adequately synthesized in the context of plant regeneration [13,14,15,16,17].

In this review, we distinguish ourselves from previous publications in three key respects: First, we propose an integrated four-layer regulatory framework that connects wound signaling, phytohormone dynamics, developmental regulators (e.g., WUS, WOX, LBD, GRF-GIF, and ARF transcription factors), and epigenetic modifications, emphasizing how these layers interact and coordinate to determine regeneration efficiency and direction. Second, we critically evaluate recent breakthroughs in regeneration technologies—particularly those aimed at breaking genotype dependence—and discuss their practical implications for crop improvement and germplasm conservation. Third, and most importantly, we explore the transformative potential of emerging cutting-edge tools, including artificial intelligence, single-cell and spatial transcriptomics, epigenome editing, and CRISPR activation systems, projecting how these technologies will drive future innovations in plant regeneration research. By bridging classical regeneration biology with next-generation technologies, this review aims to provide both a comprehensive theoretical framework and actionable insights for researchers working to overcome regeneration recalcitrance and enhance genetic transformation efficiency in plants.

## 2. Influencing Factors of Plant Regeneration

As a core biotechnology, plant tissue culture involves the in vitro culture of plant cells, tissues, or organs on artificial culture media under aseptic conditions, enabling their regeneration into complete plants [18]. The commonly used explants include cotyledons, hypocotyls, petioles, roots, microspores, and immature embryos [19]. The basic procedure involves explant sterilization, inoculation, subculture, acclimatization, and transplantation [1]. Two major pathways govern in vitro plant regeneration: organogenesis and somatic embryogenesis [20,21]. These processes involve cell dedifferentiation, proliferation, and redifferentiation, and they are regulated by multiple factors, including wound signals, plant hormones, transcriptional regulation, and epigenetic modifications [21]. Plant regeneration represents an intricate biological process governed by the coordinated action of numerous endogenous and exogenous factors [3]. The efficiency of regeneration not only depends on external conditions (such as the culture environment) but also relates to the genetic background of the plants, the physiological and developmental status of the starting explants, and the dynamic balance of hormonal signals [1].

### 2.1. Effects of Wounding on Plant Regeneration

**Figure 1:**
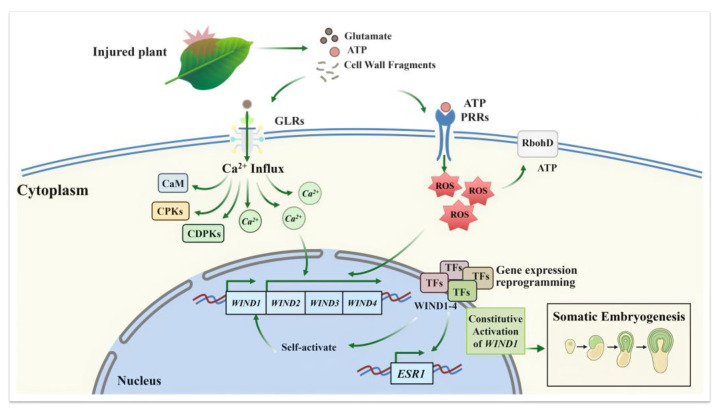
The regulatory role of wounding in plant regeneration.

Wound signaling is the initial biological event that triggers plant regeneration [22], and the signal molecules released from wounded plant tissues are recognized by pattern recognition receptors (PRRs) (including damage-associated molecular patterns (DAMPs) and microbe-associated molecular patterns (MAMPs)), thereby activating pattern-triggered immunity (PTI) [23]. Wound signals further regulate the expression of hormone-related genes, developmental genes, and epigenetic modifiers [20,24,25]. Classification of wound signals is typically based on the response kinetics of the plant, dividing them into two distinct categories [26]: The first type begins to be produced within seconds after injury and propagates rapidly throughout the plant, initiating short-term defense and stress responses—for example, changes in the electrical potential of the plant cell wall, fluctuations in calcium ion concentration, and changes in reactive oxygen species (ROS) levels [26]. The second type involves the corresponding signaling pathways activated by plant hormones, which take over from the first type to further guide the plant’s defense responses after injury [27,28] (Figure 1).

Some substances that are released when the cell is damaged are recognized by surrounding healthy cells and trigger an immediate action potential and calcium ion influx that transmit information to adjacent cells [29]. Meanwhile, high levels of reactive oxygen species are generated at the wound site, which act as a molecular signal to activate the expression of repair genes and promote cell fate transitions [30]. Glutamate, which acts as a signaling molecule, is released through the vascular system when plant leaves are wounded [31]. Existing research has revealed that glutamate can activate the glutamate receptor-like channels (GLRs) on the membranes of vascular and adjacent cells, which then trigger an influx of Ca^2+^, generating a systemic calcium wave from the wound site throughout the entire plant that is mediated by plasmodesmata and an intracellular calcium-induced calcium release mechanism [32]. Further analysis showed that the calcium wave is perceived by intracellular calcium sensor proteins, including calmodulin (CaM) and calcium-dependent protein kinases (CDPKs/CPKs), subsequently activating or inhibiting specific transcription factors and enzymes to initiate gene expression reprogramming [33,34,35] (Figure 1).

Adenosine triphosphate (ATP) is released upon the damage of leaves in *Arabidopsis*, triggering calcium signaling and the accumulation of ROS, and activating the respiratory burst oxidase homolog D (RbohD) gene, RbohD-dependent ROS propagation, and de novo root regeneration (*DNRR*) [36,37,38]. At the same time, the transcription factors of wound-induced dedifferentiation (WIND) are rapidly activated in response to injury [39]; for instance, the *WIND1–4* genes are rapidly activated upon injury and promote callus formation [40]. It has been found that WIND1 directly activates the expression of ESR1 and that its loss of function does not completely suppress callus formation, suggesting that alternative regulatory pathways exist [41,42]. Constitutive activation of WIND1 and LEC2 leads to the induction of somatic embryogenesis at both wounded and unwounded positions, which might influence regeneration by modulating cytokinin signaling [41,43].

### 2.2. Regulatory Networks of Plant Hormones in Regeneration

Plant hormones serve as pivotal regulators governing organogenesis and somatic embryogenesis, particularly the well-known auxin and cytokinin. 

#### 2.2.1. Auxin-Mediated Reprogramming and Patterning in Plant Root Regeneration

**Figure 2:**
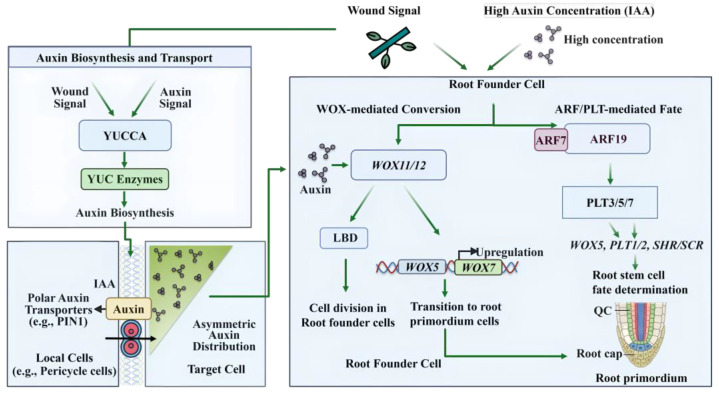
Phytohormones and developmental regulators coordinately regulate plant regeneration.

**Figure 3:**
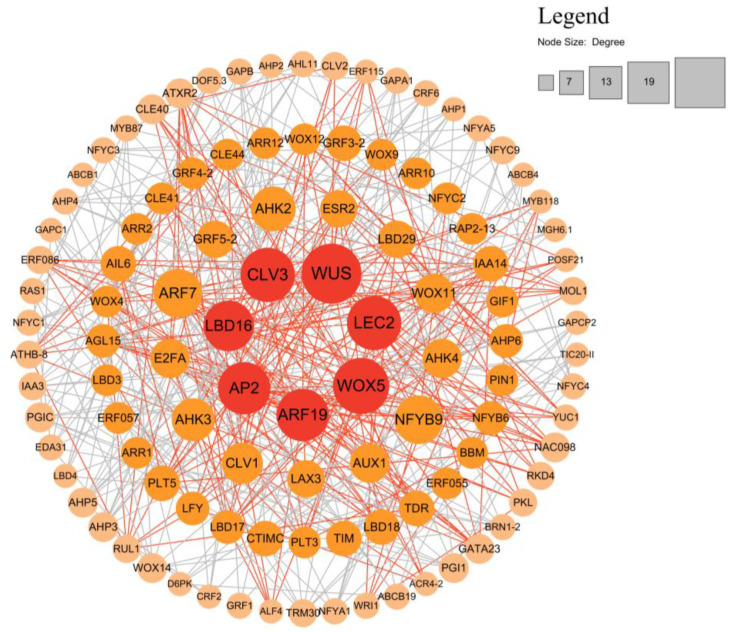
Gene interaction network. Protein–protein interaction (PPI) network of core transcriptional regulators in plant regeneration. The network was constructed using the STRING database (v12.0). Circular nodes represent individual genes and their encoded proteins, whose sizes positively correlate with connectivity degree; large dark red nodes indicate core hub genes. Red lines represent experimentally validated high-confidence direct interactions that form core regulatory modules, while thin gray lines denote predicted low-confidence indirect interactions of peripheral low-connectivity genes. The network presents a hierarchical topological structure. These core hub genes act as upstream regulators to coordinate peripheral genes, jointly modulating plant meristem homeostasis and in vitro regeneration.

Wound signals and auxin signals work together to induce specific cells to initiate reprogramming when the explants are subjected to injury or placed in a culture medium supplemented with high levels of auxin (e.g., 2,4-D); this process relies on auxin biosynthesis [44]. Studies have shown that local auxin synthesis mediated by the *YUCCA* (*YUC*) family genes is crucial for acquiring pluripotent callus (Figure 2). For example, the expression of *YUC* genes was increased after leaf abscission [26]. Subsequently, *YUC*-induced auxin was transported to cells near the wound, causing these cells to convert into specific root founder cells by inducing the expression of *WUSCHEL*-related homeobox 11 (*WOX11*) and *WOX12* [45,46,47]. *WOX11* and *WOX12* are the upstream regulators of the lateral organ boundaries domain (*LBD*) genes, which promote cell division in root founder cells [26] (Figure 3). Existing research has also found that *WOX11* and *WOX12* can upregulate the expression of *WOX5* and WOX7 by directly binding to their promoters [47,48]. Subsequently, auxin response factors (ARFs, such as ARF7 and ARF19) and PLETHORA (PLT) transcription factors (such as PLT3, PLT5, and PLT7) are also activated [49,50] (Figure 2). Once these factors are activated, the next step is to further activate downstream genes (including of *WOX5*, *PLT1/2*, and Short-root/Scarecrow (*SHR*/*SCR*)) that determine the fate of root stem cells [51]. This ultimately leads to the establishment of a functional root apical meristem, completing the de novo regeneration of the root organ [3].

It is worth mentioning that the asymmetric distribution of auxin is key to determining the site of root regeneration [52]. Polar auxin transport is essential for regeneration, which is mediated by auxin resistant1/Like-AUX1(AUX1/LAX), ATP-binding cassette subfamily B/P-Glycoprotein (ABCB/PGP), and PIN-Formed (PIN) proteins [52,53,54]. Polar transport proteins (such as PIN1) transport the auxin to specific cells and cause local auxin accumulations, thereby precisely specifying the position of root founder cells [55,56]. For example, the accumulation of auxin at the base of the stem is the direct cause of root primordium formation in *Solanum lycopersicum* [56]. However, the polarity of PIN1 is influenced by the auxin signaling output factor MONOPTEROS (MP), thereby regulating the transport and distribution of auxin itself and subsequently participating in the formation of organ patterning [57] (Figure 2).

#### 2.2.2. The Roles of Cytokinin in Regulating Plant Regeneration

Cytokinin acts as a core instructional signal in plant regeneration, particularly during *de novo* shoot regeneration [58]. Chemically synthesized cytokinin-active compounds (such as Cytokinin-Active ingreDients-A (CAD-A) and CAD-B) also have a similar function [59]. The mechanism involves a complex network comprising multi-layered signal transduction, key gene regulation, and epigenetic modifications [60]. Previous studies have shown that the cytokinin signals are first perceived by histidine kinase receptors (e.g., AHKs in *Arabidopsis*) on the cell membrane [61]; subsequently, the signal is transmitted into the nucleus through a phosphotransfer relay, ultimately activating downstream *Arabidopsis* response regulators (ARRs) [62]. 

ARR proteins are primarily classified into type-A and type-B subgroups. The typical function of type-A ARRs as negative feedback modulators, which attenuate the cytokinin signaling to prevent an overreaction of the downstream response [63]. For example, callus formation is obviously inhibited by the products of *ARR7* and *ARR15* in *Arabidopsis*. As positive regulators of cytokinin signaling, type-B ARRs are capable of directly binding to the promoters of downstream target genes (such as the stem cell key gene *WUS*), thereby initiating the regeneration program [63,64]. B-type ARR proteins predominantly drive the induction process, which is modulated by a sophisticated regulatory network [65]. For example, *ARR12* is a major enhancer of regeneration that strongly activates the stem cell regulator gene CLAVATA3 (*CLV3*) [66] (Figure 3). Conversely, *ARR1* and *ARR12* bind competitively to the promoters of *CLV3* and *WUS*, thereby counterbalancing the pro-regenerative effects of *ARR12*. Simultaneously, *ARR1* has been found to inhibit *WUS* expression by activating indole-3-acetic acid inducible 17 (*IAA17*, a repressor in the auxin signaling pathway), revealing the crosstalk between cytokinin and auxin in regeneration control [67].

Studies have confirmed that cytokinins regulate regeneration via B-ARRs and that the loss-of-function mutant B-ARRs exhibit regenerative defects, whereas their overexpression promotes callus formation [68,69]. Type-B ARRs and spatially specific HD-ZIP III transcription factors can form a complex to co-activate the expression of *WUS* in specific cells, thereby determining the site of stem cell formation within the callus [3]. The transcription of enhancer of shoot regeneration 1 (*ESR1*) can be induced by cytokinin [42]. B-type ARRs bind to the *ESR1* promoter and trigger its transcription; *ESR1* then promotes shoot regeneration by directly activating downstream target genes including CUP-shaped cotyledon 1 (*CUC1*), *WUS*, and SAM (shoot apical meristem)-related gene (*STM*) [42,70].

#### 2.2.3. Jasmonic Acid (JA) as a Stress-Responsive Coordinator in Plant Regeneration

Unlike the above mentioned classical developmental hormones auxin and cytokinin, JA does not directly drive organogenesis, instead acting more as a “two-sided coordinator” under stress conditions during plant regeneration [71] (Table 1).

The shoot regeneration efficiency was found to be significantly improved when the hypocotyls of *Arabidopsis* seedlings were pretreated with methyl jasmonate (MeJA); it was found that the promoting effect was dependent on the existence of the JA receptor protein coronatine-insensitive 1 (COI1) [72]. When plants are injured or encounter stress, jasmonic acid-isoleucine (JA-Ile) accumulates and acts as a “key” to promote the binding of the Skp1-Cullin1-F-box protein COI1 complex (SCF-COI1) with jasmonate ZIM-domain protein (JAZ) proteins [73]. This leads to the ubiquitination and degradation of JAZ and releases myelocytomatosis 2 (MYC2), which then forms a transcriptional regulatory module with co-activators (such as mediator complex subunit 25 (MED25)) and activates a series of downstream genes related to defense, cell division, and hormone homeostasis [74]. Furthermore, the signal is transmitted to the downstream transcription factor SlSBRL1 through the SlMYC2-SlPMT1/2 network, enhancing the plant melatonin signaling pathway; this forms a signal cascade that promotes adventitious root regeneration [75]. Furthermore, previous studies have also revealed that exogenous IBA must be converted to IAA to form adventitious roots [76], suggesting that JA may participate in regulating the adventitious rooting process by positively influencing the endogenous IAA levels required for this process [60,61] (Table 1).

Recent studies have consolidated the inhibitory role of JA in plant regeneration. On the one hand, when JA excessively accumulates, it overactivates defense responses at the expense of growth [77]. Exogenous JA treatment has been shown to inhibit cell-cycle progression, causing cell-cycle arrest at the G1 and G2 phases [78]. On the other hand, when JA levels are low, JAZ proteins bind to and inhibit the activity of the MYC2 transcription factor, recruiting the TOPLESS (TPL) co-repressor and preventing the expression of downstream regeneration-related genes [77]. In *Toxicodendron succedaneum*, exogenous JA inhibited adventitious root formation in a dose-dependent manner, whereas the JA biosynthesis inhibitor DIECA significantly promoted rooting [79]. Mechanistically, cytokinin-induced JA signaling represses stem cell niche genes (*PLT1* and *PLT2*) and arrests mitotic progression in *Arabidopsis* roots [80]. In *Hypericum perforatum*, high concentrations of JA (≥250 μM) suppress callus and shoot growth by inducing oxidative stress [81]. Additionally, JA inhibits rhizomes’ differentiation into shoots in orchids [82]. Taken together, the existing evidence defines JA as a condition-dependent negative modulator of organogenic processes, acting through the suppression of stem cell maintenance, cell-cycle arrest, and oxidative stress induction (Table 1).

**Table 1:** The dual role of jasmonic acid in plant regeneration. Arrows denote the direction of change in regeneration rate: ↑ increase, ↓ decrease.

Regulation	Key Molecules/Pathway	Experimental Species	Regeneration Type	Result	References
Positive	JA-Ile/COI1/MYC2 signaling cascade	*Arabidopsis thaliana*	Shoot regeneration	Shoot ↑	[73,74]
	SlMYC2-SlPMT1/2→Melatonin signaling	*Solanum lycopersicum*	Adventitious root regeneration	Root ↑	[75]
	JA→IAA accumulation (IBA conversion)	*Arabidopsis thaliana*	Adventitious root formation	Root ↑	[76]
Negative	High concentration JA (dose-dependent)	*Toxicodendron succedaneum*	Adventitious root formation	Root ↓	[79]
	Cytokinin-induced JA→Repression of PLT1/2	*Arabidopsis thaliana*	Root meristem maintenance	Root meristem ↓	[80]
	High JA (≥250 μM)→Oxidative stress	*Hypericum perforatum*	Callus and shoot growth	Callus/shoot ↓	[81]

Taken together, JA functions as a bidirectional regulator during plant regeneration, promoting shoot and adventitious root regeneration through the JA-Ile/COI1/MYC2 signaling cascade on the one hand, while suppressing regeneration at high concentrations by repressing stem cell niche genes, arresting cell-cycle progression, and inducing oxidative stress on the other (Table 1).

#### 2.2.4. Abscisic Acid (ABA) Regulates Lateral Root Development

According to recent studies, ABA also exhibits concentration-dependent bidirectional regulation in plant regeneration. On the one hand, ABA plays an inhibitory role during in vitro shoot regeneration. It has been demonstrated that exogenous ABA treatment represses the activation of *WUS* expression by suppressing histone H3 lysine 9 acetylation (H3K9ac) enrichment at specific loci of the *WUS* gene locus, thereby inhibiting stem cell niche formation and, ultimately, reducing in vitro shoot regeneration efficiency in *Arabidopsis* [83]. On the other hand, ABA promotes plant regeneration under specific conditions. In a study on somatic embryo maturation in *Pinus koraiensis*, treatment with 20 mg/L ABA combined with low-temperature pretreatment at 4 °C for two days increased the somatic embryo yield by 9.90-fold compared with the control, while also enhancing peroxidase (POD) and catalase (CAT) activities to alleviate oxidative damage [84]. Furthermore, under osmotic stress conditions, the JA pathway factor MYC2 (activated by wound signaling) and the ABA pathway factor ABI5 (activated by stress signaling) form a complex in detached leaves, which co-activates BGLU18 expression, converting inactive ABA-glucosyl ester into free ABA. This establishes a positive feedback accumulation of ABA signals, thereby enhancing the root regeneration efficiency of detached leaves under environmental stress. Exogenously applying JA and ABA in a sequential manner substantially improves the efficiency of root formation in *Arabidopsis* and poplar cuttings [85]. Notably, in a study on somatic embryogenesis in tree peony (*Paeonia ostii*), *PoSERK*, acting as a key regulatory factor, interacted with *PoABI3* to modulate BR/IAA/ABA homeostasis, thereby driving somatic embryogenesis. In watermelon, heat shock has been shown to promote storage material accumulation in unpollinated ovules by activating ABA signaling transduction, thereby increasing embryoid induction rates [86].

ABA’s bidirectional regulation also involves antagonism with gibberellic acid (GA): high ABA promotes embryo maturation genes, while GA promotes germination, with their balance determining somatic embryogenesis efficiency [87,88]. ABA and auxin exhibit complex interactions during root primordium initiation—ABA modulates auxin distribution to mediate lateral root development, while auxin feedback regulates lateral root formation by influencing ABA catabolism [89]; ABA also coordinates with ethylene to regulate cell wall remodeling and programmed cell death during root regeneration [89]. The effects of ABA are concentration-dependent: low concentrations (0.1–1 μM) promote somatic embryogenesis and root regeneration, whereas high concentrations (10–100 μM) inhibit shoot regeneration and callus proliferation [90]. This differential effect is achieved through the activation of distinct downstream networks—low ABA activates the LAFL network (LEC1-ABI3-FUS3-LEC2) to promote embryogenic competence, while high ABA suppresses cell-cycle-related gene expression [91,92]. 

In summary, the net effect of ABA depends on concentration, tissue type, and environmental conditions, mediated through complex interactions with GA, auxin, ethylene, and the LAFL network, integrating stress signals with developmental programs during plant regeneration.

#### 2.2.5. Regulatory Roles of Other Phytohormones in Plant Regeneration

In addition to auxin, cytokinin, ABA, and JA, gibberellic acid (GA) and salicylic acid (SA) also exert crucial regulatory functions during plant regeneration. GA primarily improves explant growth conditions by promoting cell elongation and division, demonstrating unique value in overcoming regeneration recalcitrance [93,94]. In highly recalcitrant spinach, the introduction of 5 µM GA_3_ following callus induction successfully stimulated shoot regeneration, achieving the first in vitro regeneration for this cultivar [95]. SA functions primarily as an antioxidant and stress-protective agent, alleviating stress-induced damage during in vitro culture [96]. In saffron (*Crocus sativus*) culture, 15 mg/L SA reduced tissue browning from 65.33% to 30.66%, shortened the culture period from 12 weeks to 6–8 weeks, and produced up to 6.57 microcorms per explant [97]. In the peach × plum hybrid rootstock *Garnem*, 50 µM SA treatment achieved a 98.33% survival rate under NaHCO_3_-induced alkaline stress [98].

In summary, GA and SA synergistically enhance plant regeneration efficiency from the dimensions of “growth promotion” and “stress protection”, respectively, offering new strategies for genetic transformation and genome editing in recalcitrant crops. 

### 2.3. Developmental Regulator-Mediated Cell Fate Reprogramming

#### 2.3.1. Involvement of the AP2/ERF Gene Family in Plant Regeneration 

The APETALA2/ethylene response factor (AP2/ERF) contains one or two DNA-binding domains that consist of 60 amino acids (known as the *AP2/ERF* domains) [99]. It is known that the different members of the AP2/ERF family have diverse functions [100]. Wound-induced dedifferentiation factor 1 (WIND1) and its paralogous genes WIND2, WIND3, and WIND4 are functionally specific members of the *AP2/ERF* family [43]. 

**Figure 4:**
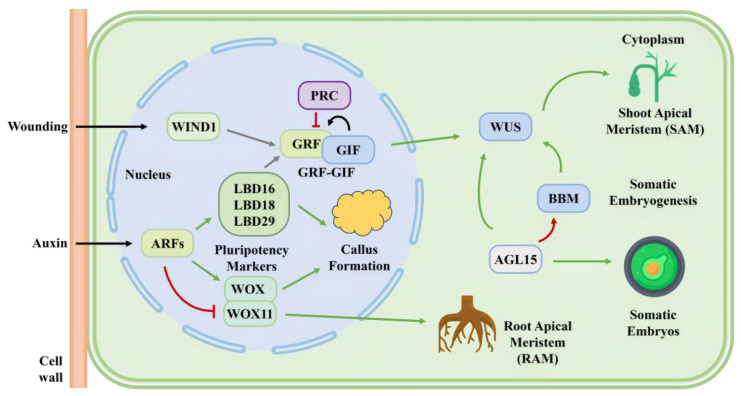
Wound signaling, phytohormones, and developmental regulators interact with one another to orchestrate plant regeneration.

Previous research has indicated that WIND1, together with its close paralogs WIND2–WIND4, promotes callus formation by activating cellular proliferation programs [101,102]. The chemically induced *AtWIND1* in *Brassica napus* and *Solanum lycopersicum*, as well as constitutively expressed *AtWIND1* in *Nicotiana tabacum*, have also been found to promote callus formation in hormone-free medium [84]. Phylogenetic analysis suggests that the WIND1-mediated signaling cascade, which promotes cell dedifferentiation, is likely conserved in at least several *Brassicaceae* and *Solanaceae* species [103]. Studies have revealed that WIND1 can also directly activate another AP2/ERF transcription factor (ESR1) to promote callus formation and shoot regeneration [104,105]. The *esr1* mutant exhibited defects in both callus formation and *de novo* shoot regeneration, whereas overexpression of *ESR1* can promote both processes [42]. Simultaneously, ESR2 can fine-tune cytokinin homeostasis by directly regulating cytokinin biosynthesis genes (such as *IPT5*), thereby affecting green callus formation and vascular development [106]. Furthermore, WIND1 induction can trigger somatic embryogenesis even in hormone-free medium [22], and it was revealed that WIND1 can modulate various developmental programs to boost the regeneration process [22]. At the cellular level, WIND1 can promote regenerative efficiency by bypassing the wounding and the early incubation step in auxin-rich callus-inducing medium (CIM), while cytokinin-inducing medium (SIM) is the typical prerequisite for regeneration [107] (Figure 4).

BABY BOOM (BBM), another AP2/ERF family member, is a well-known master regulator of somatic embryogenesis [108]. Ectopic expression of BBM induces spontaneous somatic embryo formation without exogenous hormone treatment in *Arabidopsis* and *Brassica napus* [108]. BBM functions by activating downstream genes involved in auxin biosynthesis, cell wall remodeling, and stress responses [109]. Notably, BBM has been successfully deployed to improve transformation efficiency in recalcitrant crops, including maize, wheat, and soybeans, often in combination with GRF-GIF or WUS2 [110,111].

Several other AP2/ERF family members have also been found to be involved in regeneration; for example, *PLT3/5/7* are indispensable for wound-induced callus formation and pluripotency acquisition under CIM/SIM culture conditions, activating the root stem cell regulators *PLT1* and *PLT2*, and promoting the shoot factor *CUC2* to complete the shoot regeneration process [112]. Research has revealed that *PUCHI* is crucial for regulating cell division patterns, participating in lateral root initiation and development by modulating auxin signaling [113,114]. The wound-inducible *ERF115*, which controls root stem cell reorganization after injury, acting upstream of WIND1, is a rate-limiting factor for cell division in the quiescent center (QC) of roots [115]. ERF113/RAP2.6L, a close homolog of ERF115, has also been reported as a key regulator of tissue reunion and as a regeneration regulator under CIM/SIM conditions [107].

#### 2.3.2. Accelerating Plant Regeneration with GRF-GIF

Growth-regulating factor (GRF) and GRF-interacting factor (GIF) are plant-specific transcription factor families. GRF proteins are capable of modulating target gene expression through direct DNA binding, whereas GIF proteins exert their functions by interacting with GRFs to assemble a functional transcriptional complex known as the GRF-GIF complex, which serves as a crucial regulator in the cellular reprogramming process underlying plant regeneration [116] (Table 2). 

It has been revealed that the *TaGRF4-GIF1* complex in wheat can elevate the transformation efficiency significantly, drastically shortening the regeneration period while also increasing the number of transformable wheat genotypes [117]. Co-expression of *GRF4-GIF1* and overexpression of *GRF5* have also been found to enhance the genetic transformation efficiency significantly in *Sorghum bicolor* [118]. The transformation efficiency in *Zea mays* was increased 3.5 to 6.5 times by co-expression of *ZmGRF1-ZmGIF1* [119]. Similar functions of the *GRF-GIF* complex exist in dicotyledonous plants; for example, in *Glycine max*, the regeneration and transformation efficiency improved significantly, and the number of transformable varieties was also increased by overexpression of the GRF3-GIF1 complex [120]. Other members of the GRF-GIF protein family have likewise been successfully utilized to boost plant transformation efficiency [120]; for example, the transformation efficiency of *Beta vulgaris*, *Glycine max*, *Helianthus annuus*, *Zea mays*, and tomato was improved by the heterologous expression of *AtGRF5* [121]. Similarly, GRF4-GIF1 from *Solanum lycopersicum*, *Vitis vinifera*, and *Citrus* have all been demonstrated to improve transformation efficiency in *Lactuca sativa* [122]. 

From the above findings, it can be inferred that the GRF-GIF complex possesses wide applicability among diverse genotypes and displays no obvious functional discrepancies across distinct plant species.

#### 2.3.3. Diverse Functions of WOX in Enhancing Plant Regeneration Efficiency

The WOX gene family exerts a vital regulatory effect on plant growth and development [123], which is indispensable for the formation and maintenance of the stem cell niche during shoot regeneration [64] (Table 2). 

In the woody plants *Populus trichocarpa* and *Betula platyphylla*, *PtrWOX4* and *BpWOX4* can promote cambial cell proliferation, respectively [124,125]. *WOX4* homologs in *Malus domestica* and *Larix gmelinii* have demonstrated functions in enhancing organogenesis [126]; for instance, the *de novo* regeneration in transgenic leaf explants during *in vitro* culture was enhanced by overexpression of *MdWOX4* in *Nicotiana tabacum*, although additional hormones were required [126]. Similarly, the natural root formation in *Populus trichocarpa* cuttings was accelerated by overexpression of *LkWOX4* [127]. Overexpression of *MiWOX1b*, *4a*, and *4b* in *Macadamia* was also found to enhance *de novo* root and callus regeneration significantly [128]. It was found that *ScWOX4/9/10/12* were upregulated during callus proliferation and shoot regeneration in *Saccharum officinarum* [129]. In *Triticum aestivum*, it was also found that the regeneration efficiency was enhanced by overexpression of *TaWOX1*, *TaWOX5*, *TaWOX9*, and *TaWOX14*, with *TaWOX14* showing the most significant effect [130]. Heterologous overexpression of *PoWOX1* from *Paeonia ostii* in *Arabidopsis* can promote root growth, early shoot emergence, flowering, and more frequent divisions in pericyclic cells adjacent to the xylem in *PoWOX1*-OE lines [131]. Thirteen WOX genes in *Ginkgo biloba* were identified (designated as *GbWUS* and *GbWOXs*), showing that *GbWOX2* was specifically expressed in embryos and promoted callus induction, while *GbWOX3A* showed preferential expression during early stages of embryo and callus development [132], and overexpression of *GbWOX2* in *Paeonia ostii* and *Solanum lycopersicum* resulted in larger and denser callus formation [132]. Meanwhile, *GbWOX3A* substantially improved shoot regeneration efficiency and significantly increased the adventitious bud induction efficiency [132].

Furthermore, transformation involving WOX family genes may cause phenotypic defects in transgenic lines [19]; for example, overexpression and knockdown of *NsWOX9* in *Nicotiana sylvestris* led to leaf deformities, indicating its important role in leaf development [133]. Overexpression of *AtWOX1* resulted in meristem abnormalities, subsequently leading to small leaves, reduced fertility, and dwarfism in *Arabidopsis* plants [134,135]. In *Medicago truncatula* and *Nicotiana sylvestris*, accurate leaf morphogenesis and patterning depend on direct transcriptional repression of *WOX9* by the *WUS*-clade regulators STF and LAM1 [136]. 

Collectively, these findings reveal that direct interactions between activating and repressing *WOX* genes function to maintain the homeostatic balance between cell proliferation and differentiation. This regulatory module may represent an evolutionarily conserved mechanism governing the complex and diversified morphological development in flowering plants [136] (Figure 4). 

#### 2.3.4. LBD-Mediated Regulatory Networks in Auxin-Driven Plant Regeneration and Development

Functional identification of different LBD members indicates that these genes are key regulators in defining the boundaries of lateral organs and governing multiple developmental processes in plants [137] (Table 2). It was found that callus induction during *Arabidopsis* regeneration is mediated by LBD genes, and that some LBD genes (for example, *LBD16*, *LBD17*, *LBD18*, and *LBD29*) downstream of ARFs are highly induced by CIM [138]. Previous studies have shown that these *LBD* genes can contribute to callus development during *in vitro* plant regeneration by mediating auxin signaling [138], and further analysis indicated that the gene products of *ARF7* and *ARF19* promote *in vitro* callus formation by upregulating these *LBD* genes [139] (Figure 4). 

It was found that *LBD13* can regulate lateral root formation, located in the nucleus and expressed in emerged lateral roots (LRs) and the LR meristem of elongating lateral roots in *Arabidopsis* [140]. A total of 73 *PgLBD* genes were identified in *Panax ginseng*, and *PgLBD18* and *PgLBD49* were found to be the key regulators of lateral root development, mediating auxin signaling to regulate lateral root development via the *PgARF-PgLBD* module [141]. Similarly, a genome-wide *LBD* family identification was also conducted in *Malus domestica*, and it was found that *MdLBD16a* is significantly upregulated during adventitious root formation after auxin treatment [142](Figure 4). 

However, leaves curling upward and failure to expand properly were found in *LBD*-overexpression lines in *Arabidopsis* [143]. Similar phenomena have also been observed in *Oryza sativa*, with reduced leaf size and number of bulliform cells, accompanied by sparse and inward leaf curling, in lines overexpressing the *LBD3* gene; this suggests that *OsLBD3-7* potentially act as upstream regulators of bulliform cell development [144].

#### 2.3.5. Additional Key Regulators: SERK and AGL15

SOMATIC EMBRYOGENESIS RECEPTOR-LIKE KINASE (SERK) family members are leucine-rich repeat receptor-like kinases (LRR-RLKs) that serve as essential signaling components for embryogenesis competence [145]. SERK1 was originally identified in carrots as a marker for embryogenic cells [145]; in *Arabidopsis*, *AtSERK1* expression is enriched in embryogenic cells, and its overexpression enhances somatic embryogenesis efficiency [145]. Importantly, SERK proteins function as co-receptors in brassinosteroid and embryogenesis signaling pathways, forming complexes with BRASSINOSTEROID INSENSITIVE 1 (BRI1) and other receptor kinases [146,147]. Phylogenetic analysis has revealed that SERK homologs are conserved across diverse plant species, including maize, rice, and woody plants, suggesting a universal role in conferring embryogenic competence [148,149]. The molecular link between SERK-mediated signaling and downstream transcriptional regulators such as BBM, LEC1, and WOX family members remains an active area of investigation [150,151].

AGAMOUS-LIKE 15 (AGL15) is a MADS-box transcription factor that promotes both somatic embryogenesis and shoot regeneration. AGL15 is highly expressed during early embryogenesis, and its ectopic expression enhances somatic embryo formation in *Arabidopsis*, *Medicago truncatula*, and other species [152,153]. Mechanistically, AGL15 directly binds to the promoters of embryogenesis-related genes, including *BBM*, *LEC1*, *LEC2*, and FUS3, while also interacting with chromatin remodelers to establish a transcriptionally permissive environment [154,155]. Recent evidence indicates that AGL15 influences histone acetylation by recruiting HATs to target loci, while also functioning synergistically with other developmental regulators; for example, co-expression of AGL15 and BBM exhibits additive effects in promoting regeneration [152,156,157].

**Table 2:** The roles of developmental regulatory factors in plant regeneration.

Gene Family	Function	Representative Factors	Example	References
AP2/ERF	Wound response and cell dedifferentiation	WIND1, ESR1, ERF115	*Arabidopsis*, Tobacco	[113,158]
GRF-GIF	Cellular reprogramming and growth control	GRF4-GIF1, GRF5	Wheat, Sorghum, Maize, Citrus, Soybean	[117,122]
WOX/WUS	Stem cell niche establishment and maintenance	WUS, WOX4/5/11/12	*Arabidopsis*, Wheat, Poplar, Sugarcane	[117,124,131]
LBD	Callus induction and organogenesis initiation	LBD16/17/18/29	*Arabidopsis*, Apple, Ginseng, Rice	[140,144]
Other important regulators	Multi-pathway synergistic regulation	PLT1/2/5/7 CUC, TCP3/4	*Arabidopsis*	[112,159]

### 2.4. Epigenetic Dynamic Regulation and Plasticity During Regeneration

Epigenetic mechanisms, including chromatin accessibility, histone modifications, DNA methylation, and RNA silencing, make critical contributions to cell dedifferentiation and redifferentiation [160,161,162,163].

#### 2.4.1. Chromatin Accessibility in Transcriptional Activation

Chromatin is a complex of DNA and histones, whose state determines DNA accessibility. It has been found that a transient and global increase in chromatin accessibility occurs at the initial period of callus generation, laying the foundation for cellular reprogramming [164]. This is followed by the activation of core reprogramming factors; for instance, auxin signaling induces the chromatin remodelers (such as the BRM protein of the SWI/SNF family) and the histone acetyltransferases (HATs) to bind to the promoter regions of key embryogenic or stem cell genes (e.g., *LEC1*, *LEC2*, *WOX5*, *PLT*) [165], which open the chromatin in these regions by adding activating histone marks (e.g., H3K4me3) and evicting nucleosomes, and by switching the genes from a silent state to an activatable one [166,167]. The SWI/SNF chromatin remodeling complex, as a core executor, mobilizes or evicts nucleosomes through ATP hydrolysis, which is fundamental for increased chromatin accessibility [168]. Meanwhile, the chromatin of genes unrelated to the original meristems (e.g., those specific to leaves and roots) is inactivated by histone deacetylases (HDACs) and repressive histone modifications (e.g., H3K27me3), promoting cells to exit their original differentiation programs [166,167].

#### 2.4.2. Histone Acetylation Dynamics Orchestrate De Novo Root Regeneration

Histone modifications are typically involved in acetylation, methylation, phosphorylation, and other processes. Methylation events (mono-, di-, tri-methylation) occur at lysine and arginine sites on histone tails; these alterations reshape the local chromatin configuration, further regulating the transcriptional capacity of corresponding chromosomal segments [164,169,170,171].

In *Arabidopsis*, the epigenetic mark H3K27me3, i.e., histone H3 lysine 27 trimethylation, is enzymatically catalyzed by polycomb repressive complex 2 (PRC2), and it has been found that the loss of PRC2 function can promote somatic embryogenesis from vegetative tissues [172]. Functionally antagonistic to PRC2 are the Trithorax Group proteins (TrxG), which counteract PRC2-mediated repression by depositing activating marks such as H3K4me3, thereby ensuring accurate spatiotemporal gene activation [173,174]. DNA methylation and H3K27me3 demethylation may activate genes such as *PpLBD1* and *PpPIN6*, thereby inducing callus formation when leaf explants were used for culture experiments in *Prunus persica* [175]. The callus induction rate in peach significantly decreased after treatment with GSK-J4 (an inhibitor of H3K27me3 demethylase) [175]. During root apical meristem development in rice, PLT3/4/5 recruit PRC2 and JMJ703 to increase the H3K27me3/H3K4me3 ratio at differentiation-associated loci, thereby maintaining stem cell identity [176]. Wound signaling activates the auxin synthesis gene anthranilate synthase alpha subunit 1 (*ASA1*) via ERF109, and this rapid activation depends on pre-existing H3K36me3 modifications that prime *ASA1* for quick responses to promote root regeneration [177]. Additionally, HDAC inhibitors elevate H3K9/K14ac levels at key shoot regeneration loci, activating their expression and promoting shoot regeneration in *Arabidopsis* and wheat [178].

Wounded leaf vascular tissues initiate de novo root formation on hormone-free MS medium; the underlying mechanism involves *ESR1* recruiting the histone deacetylase6 (HDA6) to the JAZ5 locus, and the expression of JAZ5 is repressed by HDA6-mediated histone H3 deacetylation [158]. ERF109 activates the key enzyme ASA1 in the biosynthesis of IAA with the inhibitory effect of JAZ5 [158]. However, when wounded leaves are placed in auxin-rich CIM, the exogenous 2,4-D inhibits the IAA biosynthesis within the vascular tissue through HDA6-dependent histone deacetylation and repression of *YUC1*, *YUC7*, and *YUC9* [179]. Local auxin accumulation at the wound site subsequently triggers root founder cell specification and de novo root regeneration [180,181].

#### 2.4.3. DNA Methylation Orchestrates Callus Formation and Organogenesis

As a crucial epigenetic modification, DNA methylation is sustained by diverse DNA methyltransferases, including MET1 (also designated DMT1), CMT3, DRM2, and CMT2 [182,183]. 

**Figure 5:**
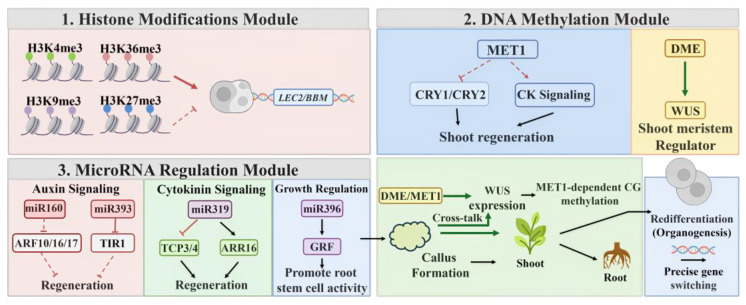
Module of epigenetic modification in plant regeneration.

When plant somatic cells are induced by exogenous hormones, they need to detach from their differentiated state and revert to a stem cell-like state to form callus [184]. It has been reported that the extent of global DNA methylation was decreased significantly during the initial stages of callus induction; this global “erasure” helps to dismantle the original identity of the somatic cells and endow the cells with developmental plasticity [185]. Crucial genes involved in stem cell self-renewal and meristem function (e.g., *WUSCHEL*, *WOX5*) undergo specific demethylation in their promoter regions, leading to their activation and expression, and thereby initiating the callus program [3]. Genes that promote callus characteristics are remethylated and silenced, ensuring that cells transition from a proliferative state to organized organogenesis [186]. The differentiation-related genes are silenced through hypermethylation [187], and it has been found that the genome undergoes remethylation at new sites when callus is directed to form new organs under appropriate hormone ratios [188]. Meanwhile, the precise regulation of key developmental genes is also required; for example, the expression of *WUS* needs to be precisely confined to specific regions during shoot regeneration [189]. This precise spatial expression pattern is partially achieved by maintaining hypomethylation (activation) in the promoter region within specific cells while ensuring hypermethylation (silencing) in other areas [190,191] (Figure 5).

It has been demonstrated that the shoots of *Arabidopsis cmt3* mutant (which is lacking in the CHG methylation chromomethylase 3) exhibited high regenerative capacity on SIM and could undergo direct organogenesis [192]; it was also found that the blue light receptor gene CRYPTOCHROME 1 (*CRY1*) is upregulated to facilitate shoot regeneration in the *met1* mutant [193]. Further analysis showed that both *cmt3* and *met1* mutants exhibited enhanced regeneration rates [51,192,194]. The RNA-directed DNA methylation (RdDM) pathway plays a unique role in this process, using small RNAs to guide the DNA methyltransferase DRM2 to carry out de novo methylation at homologous sequences. Studies have shown that LEC2 also promotes somatic embryogenesis by inducing CHH hypermethylation through the RdDM pathway [195]. DNA hypomethylation has also been demonstrated to facilitate callus generation and adventitious root regeneration in peach and black locust [175], and treatment with the DNA methylation inhibitor 5-azacytidine dramatically enhances callus formation, which provides additional evidence for the importance of DNA methylation in plant regeneration [175]. This indicates that, beyond the “open” state of global demethylation, precise, locus-specific de novo methylation is also critical for the successful completion of reprogramming. 

#### 2.4.4. The Regulatory Role of microRNAs in Plant Regeneration 

The regulatory processes of cell reprogramming and organ regeneration are critically modulated by microRNAs, which act as key post-transcriptional regulators by tuning the expression of their corresponding target genes [196,197]. 

High levels of miR156 in the juvenile phase of *Arabidopsis* seedlings repress the expression target gene of SQUAMOSA promoter binding protein-like 9 (*SPL9*) [198], which can bind to type-B ARRs and inhibit the cytokinin response, thereby attenuating shoot regeneration capacity [199]. As a small RNA methyltransferase, the protein encoded by HUA enhancer 1 (*HEN1*) is essential for normal cytokinin responses; consequently, *hen1* mutations trigger cytokinin-related defects during de novo shoot regeneration [200]. Loss-of-function mutations in *HEN1* cause a marked reduction in mature miR319 levels, relieving post-transcriptional repression of its target genes *TCP3* and *TCP4*. The resulting accumulation of TCP3/TCP4 proteins not only suppresses de novo shoot regeneration but also directly activates the transcription of *ARR16*, thereby dampening the cytokinin signaling output [164,200]. Taken together, these findings indicate that the miR319-*TCP3/4*-*ARR16* regulatory cascade controls de novo shoot regeneration via adjusting cytokinin response pathways [200] (Figure 5).

miR160s are expressed in vascular tissues, with their mature miRNA molecules moving to meristematic cells to target and break down *ARF10* and *ARF17* messenger RNAs, thereby de-repressing *BRAVO* and *WOX5* transcription that was previously inhibited by ARF10 and ARF17 [201,202]. Consequently, *BRAVO* cooperates with *WOX5* to maintain the quiescent state of QC cells and stem cell pluripotency [201,202]. It has been found that *ARF10/17* accumulates in QC cells and subsequently downregulates *BRAVO* and *WOX5* gene expression when miR160 levels decrease, ultimately prompting these QC cells to divide and proliferate to replenish damaged stem cells for root regeneration [202]. Additional research indicated that the application of an miRNA-resistant ARF17 construct led to an increase in ARF17 mRNA abundance and, concurrently, the accumulation of auxin-inducible GH3-like messenger RNAs (YDK1/GH3.2, GH3.3, GH3.5, and DFL1/GH3.6) underwent changes [203]. These expression changes cause growth defects such as abnormal symmetry in embryos and nascent leaves, as well as leaf shape defects [203,204]; these abnormalities highlight the critical role of miR160-mediated regulation on *ARF17* [203,205,206]. It should be noted that the precise mechanisms by which miRNAs modulate callus initiation and formation are yet to be fully understood.

#### 2.4.5. Crosstalk Between Transcription Factors and Epigenetic Regulators

Plant regeneration is a complex developmental process that requires intricate coordination between transcription factors and epigenetic regulators. This synergy ensures precise spatiotemporal control of gene expression. On the one hand, transcription factors act as “commanders” that directly recruit specific epigenetic modifying enzymes to target gene loci. For example, the auxin response factor ARF5 (MP) has been shown to directly interact with BRM, a subunit of the SWI/SNF chromatin remodeling complex, to co-activate the expression of LEC1, a key gene for embryogenesis initiation [176,207]. On the other hand, epigenetic modifications can reciprocally influence the binding ability of transcription factors. For instance, a closed chromatin state (e.g., regions enriched with H3K27me3) prevents transcription factor binding; only after H3K27me3 is removed by demethylases such as JMJ703 can the relevant developmental transcription factors access and initiate transcription [176]. Similarly, the aforementioned cases of ESR1 recruiting HDA6 to the JAZ5 locus [158] and ERF109 depending on the pre-existing H3K36me3 modification at the ASA1 locus are both typical examples of transcription factor–epigenetic modifier synergy. This “transcription factor–epigenetic modification” interaction network tightly couples external hormonal signals with the nuclear machinery that reads, writes, and erases epigenetic marks, representing the core molecular basis for cell fate transition [208].

### 2.5. Cross-Species Comparison of Plant Regeneration: Conservation and Specificity

Beyond the empirical observations of species- and genotype-dependent regeneration, a systematic comparison across plant taxa reveals that regeneration is governed by both conserved principles and lineage-specific adaptations. Regarding monocots versus dicots, the classical two-step regeneration model was largely established in dicot model species, particularly *Arabidopsis thaliana* [209]. When applied to monocot crops such as rice, maize, and wheat, this model requires substantial adjustments in hormone types, concentrations, and treatment durations, reflecting fundamental differences between the two lineages in auxin biosynthesis, polar transport, and ARF/Aux/IAA-mediated transcriptional responses. Cytokinin signaling components also exhibit lineage-specific expression patterns that influence shoot regeneration competence [209]. These differences indicate that while the regulatory logic of stem cell maintenance—exemplified by the WUSCHEL-CLAVATA feedback loop—is conserved between monocots and dicots, its molecular implementation has undergone lineage-specific divergence [209]. Regarding herbaceous versus woody plants, herbaceous species, characterized by rapid growth and short life cycles, generally display higher regenerative plasticity [210]. In contrast, woody plants face additional constraints imposed by secondary growth, lignification, phenolic compound accumulation, and ontogenetic phase changes, resulting in a marked decline in regeneration capacity with donor age—a phenomenon tightly linked to the miR156/SPL age pathway [11,211]. The more stable DNA methylation and histone modification landscapes in woody plants render their somatic cells more resistant to the extensive chromatin remodeling required for pluripotency acquisition. Moreover, wound-induced callus formation in woody species is typically slower and more localized, suggesting that the propagation of systemic wound signals—including Ca^2+^ waves, reactive oxygen species bursts, and jasmonate/ethylene cascades—is constrained in woody perennials [28,212,213]. Regarding model species versus recalcitrant crops, model species such as *Arabidopsis* and rice possess small genomes, low transposable element contents, and labile epigenetic configurations that facilitate cellular memory erasure and fate reprogramming [214,215]. In contrast, recalcitrant crops—including soybeans, peas, wheat, and barley—have large, complex genomes with high repeat content and stable methylation landscapes, which create formidable epigenetic barriers [164,216]. Such recalcitrance can only be partially overcome through specific genotype–medium combinations; the use of juvenile tissues; or the overexpression of regeneration-enhancing transcription factors such as *TaWOX5*, *BBM*, and *GRF* chimeras, indicating that species-specific regulatory factors can serve as master switches to unlock otherwise epigenetically silenced regeneration potential [108,117,217].

Synthesizing the above comparisons, the regulatory mechanisms of plant regeneration can be categorized into relatively conserved mechanisms and species-specific mechanisms. Conserved mechanisms include wound-induced local auxin biosynthesis and polar transport as the initial trigger for pluripotency establishment, the central role of ARF-mediated transcriptional cascades in reprogramming, the fundamental function of the WUSCHEL/WOX family in stem cell homeostasis maintenance, and the universal involvement of chromatin remodeling mediated by H3K27me3/H3K4me3 dynamics in cell fate transitions [64,199,218,219]. Species-specific mechanisms include the identity and hierarchy of transcription factors that serve as competence regulators for regeneration across different species (e.g., PLETHORA is critical in *Arabidopsis* but may be substituted by other families in monocots) [111,112]; interspecific differences in the precise hormonal thresholds and synergistic/antagonistic interactions among auxin, cytokinin, and other phytohormones; variation in the genomic targets of DNA demethylases and histone modifiers shaped by lineage-specific genome composition and transposable element distribution [220]; and differential contributions of non-cell-autonomous signals such as mobile small RNAs or peptide hormones across species [203,221]. 

In conclusion, plant regeneration can be conceptualized as a conserved “core circuit” of wound–hormone–epigenetic interactions, upon which species and lineage-specific “modulatory layers” have been superimposed during evolution. Distinguishing core circuit components from modulatory layers is not only of theoretical significance but also has direct practical value for the rational design of regeneration protocols in recalcitrant species.

## 3. Conclusions and Perspectives

Plant regeneration is an intricate biological event coordinated by multiple internal and external signals [208,222]. Wound signaling serves as the initial trigger for regeneration, inducing local damage responses that activate the accumulation of downstream defense hormones such as JA and initiate a series of transcriptional reprogramming events [177,223]. The dynamic balance of phytohormones precisely regulates the direction of root/shoot differentiation [224,225,226]. Developmental regulators (e.g., WUS, WOX, GRF-GIF, LBD transcription factors) are core executors of cell fate determination, directly activating or repressing downstream target genes to confer totipotency or guide differentiation toward specific organs [64,120,218,227]. Epigenetic modifications dynamically regulate the accessibility of regeneration-associated genes, ensuring precise transcriptional reprogramming during dedifferentiation and redifferentiation [161,178,187,228,229]. These four layers form an intricate regulatory network that collectively determines the efficiency and direction of plant regeneration.

Advances in regeneration technology are enabling genome editing to overcome the traditional bottleneck of genotype dependence. To address the issue of crop recalcitrance, CRISPR/Cas-mediated genetic transformation can be integrated to ameliorate poor regeneration efficiency [15,230,231]. Based on an in-depth understanding of somatic embryogenesis and epigenetic regulatory mechanisms, efficient in vitro regeneration technologies provide new solutions for the conservation of endangered plants [232,233]. Meanwhile, precise manipulation of hormone regulatory networks and developmental regulators enables the establishment of full process-efficient systems from callus induction to rooting acclimatization, providing core technical support for targeted breeding [16,234]. 

It is worth noting that emerging technologies—represented by artificial intelligence, single-cell multi-omics, epigenome editing, and CRISPR-mediated activation—are profoundly reshaping the research paradigm in the field of plant regeneration.

In the area of artificial intelligence-assisted prediction, predictive models based on deep learning architectures such as multi-layer perceptron (MLP) have achieved high-precision prediction of regeneration potential in woody plants by integrating multidimensional variables, including environmental, ecological, and forestry parameters, thereby providing novel strategies for optimizing regeneration conditions and screening highly regenerative germplasms [235]. Meanwhile, the application of single-cell transcriptomics (scRNA-seq) enables researchers to dissect the dynamic developmental trajectory of callus formation at the cellular level [236]. Through single-cell sequencing of *Arabidopsis* callus at different stages (initiation, proliferation, greening, and shoot differentiation), researchers have identified key transcription factor networks involved in the formation of root and shoot primordia and revealed the regulatory effects of environmental factors, such as hypoxia and salt stress, on callus initiation [13,237]. Spatial transcriptomics goes a step further by mapping gene expression profiles during regeneration while preserving spatial positional information. A recent study on poplar demonstrated that spatial transcriptomics can trace (at high resolution) the differentiation path from cambium cells to root primordium cells after wounding, reveal the sophisticated spatial dialogue between auxin and cytokinin signaling pathways, and identify novel molecular markers such as SAC56 and LOS1 that promote root regeneration [14]. The combined application of single-cell and spatial transcriptomics is driving plant regeneration research from static gene list descriptions toward dynamic, high-resolution, four-dimensional regulatory network dissection.

At the level of epigenetic regulation, the development of epigenome editing technologies has provided unprecedented tools for manipulating cell fate reprogramming. Studies have revealed that the transcription factor LEC2 increases chromatin accessibility and activates the expression of totipotency-related genes through the RdDM pathway [185]. This finding breaks through the technical bottlenecks of traditional embryonic research systems and provides important theoretical support for improving crops’ genetic transformation efficiency via epigenetic modification. Concurrently, researchers have developed a chemically inducible CRISPR activation (CRISPR-a) tool called the ER-Tag system, which combines the XVE induction system with the SunTag CRISPR-a system to achieve temporally specific activation of endogenous regeneration-associated genes [238]. In multiple plant species, including alfalfa, woodland strawberry, and sheepgrass, induced activation of developmental regulator genes (e.g., morphogenic genes) significantly accelerated the regeneration process and improved regeneration efficiency [238]. This strategy provides a powerful platform for studying the functions of genes that are lethal or difficult to transform when constitutively expressed, as well as for large-scale screening of effective morphogenic gene pairs [238].

In terms of genotype-independent transformation strategies, researchers have established a breakthrough technology system using soybeans as a model. The GiFT (Genotype-independent Fast Transformation) method uses germinating seeds as explants combined with *Agrobacterium* infection and in vivo selection, minimizing tissue culture manipulation and enabling the recovery of healthy transgenic plants within approximately 35 days [239,240]. This method has been successfully applied to diverse elite germplasms, is compatible with CRISPR/Cas12a vectors, and achieves high heritability of transgenes in the T1 generation. This strategy provides a transferable paradigm for overcoming genotype-dependent bottlenecks in major crops and enabling high-throughput genetic transformation and gene editing in breeding [17].

It is foreseeable that, as our understanding of plant regeneration regulatory networks deepens and cutting-edge technologies become further integrated, plant regeneration will evolve from a traditional seedling propagation technique into a core driving force of new agricultural productivity encompassing germplasm innovation and efficient propagation [10,15,230].

## Data Availability

No new data were created or analyzed in this study. Data sharing is not applicable to this article.
